# Supplementary material for: Gapless spin liquid in a square-kagome lattice antiferromagnet
Source: Nat Commun. 2020 Jul 9;11:3429. doi: 10.1038/s41467-020-17235-z (PMC7347939; doi:10.1038/s41467-020-17235-z)
Supplement: Supplementary file 1 — Supplementary Information [file 41467_2020_17235_MOESM1_ESM.pdf]

Gapless spin liquid on square-kagome lattice

Fujihala *et al.*

Gapless spin liquid on square-kagome lattice

M. Fujihala[1,*], K. Morita[2,†], R. A. Mole[3], S. Mitsuda[1], T. Tohyama[2], S. Yano[4], D. H. Yu[3], S. Sota[5], T. Kuwai[6], A. Koda[7], H. Okabe[7], H. Lee[7], S. Itoh[8], T. Hawai[8], T. Masuda[9], H. Sagayama[10], A. Matsuo[11], K. Kindo[11], S. Ohira-Kawamura[12], and K. Nakajima[12]

[1]Tokyo University of Science, Department of Physics, Tokyo, 162-8601, Japan
[2]Tokyo University of Science, Department of Applied Physics, Tokyo, 125-8585, Japan
[3]Australian Nuclear Science and Technology Organisation, Lucas Heights, New South Wales 2232, Australia
[4]National Synchrotron Radiation Research Center, Hsinchu 30077, Taiwan
[5]Computational Materials Science Research Team, RIKEN Center for Computational Science, Kobe, Hyogo 650-0047, Japan
[6]Graduate School of Science and Engineering, University of Toyama
[7]Muon Science Laboratory and Condensed Matter Research Center, Institute of Materials Structure Science, High Energy Accelerator Research Organisation, 1-1 Oho, Tsukuba 305-0801, Japan
[8]Neutron Science Division, Institute of Materials Structure Science, High Energy Accelerator Research Organisation, 1-1 Oho, Tsukuba, Ibaraki 305-0801, Japan
[9]Institute for Solid State Physics, The University of Tokyo, Kashiwa, Chiba 277-8581, Japan
[10]Synchrotron Radiation Science Division 1 and Condensed Matter Research Center, Institute of Materials Structure Science, High Energy Accelerator Research Organisation, 1-1 Oho, Tsukuba, Ibaraki 305-0801, Japan
[11]International MegaGauss Science Laboratory, Institute for Solid State Physics, The University of Tokyo, Kashiwa, Chiba 277-8581, Japan
[12]Materials and Life Science Division, J-PARC Center, Tokai, Ibaraki, 319-1195, Japan

*fujihara@nsmsmac4.ph.kagu.tus.ac.jp
†katsuhiro.morita@rs.tus.ac.jp

Supplementary Information

Supplementary Note 1: Crystal structure refinement

The synthesis of $KCu_6AlBiO_4(SO_4)_5Cl$ was motivated by the identification of the naturally occurring mineral atlasovite, $KCu_6FeBiO_4(SO_4)_5Cl$ [Supplementary Reference 1]. The crystal structure of the obtained polycrystalline $KCu_6AlBiO_4(SO_4)_5Cl$ is investigated by synchrotron X-ray powder diffraction with wavelength of 0.68892 Å at BL-8B, Photon Factory and the crystal parameters were refined by the computer program RIETAN-FP [Supplementary Reference 2]. The space group for $KCu_6AlBiO_4(SO_4)_5Cl$ is *P*4/*ncc* which is same as that of atlasovite (Fe-site is replaced by Al^{3+} ions). The lattice parameters are determined to be a = 9.8248(9) Å and c = 20.5715(24) Å, respectively. The positions of the individual atoms are also determined as presented in TABLE I (the occupancies of atom were fixed to 1.0, isotropic atomic displacement parameters B was fixed to 0.5 or 1.0.). The low R factors and goodness-fit-indicator S indicate the satisfactory refinement for this compound.

In order to check the Cu site cleanliness, we attempted to refine the Cu defect model (the occupancies of atoms were fixed to 1.0 , excluding that of Cu). If the Cu is replaced by Al, Bi, K, the occupancy of Cu should be deviated from 1.0. The occupancies of Cu1 and Cu2 sites are refined to 1.004(8) and 0.965(12), indicating the Cu sites have no defects in $KCu_6AlBiO_4(SO_4)_5Cl$.

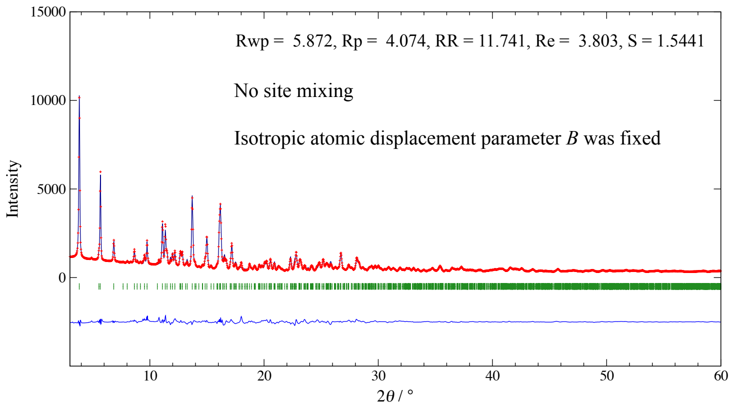

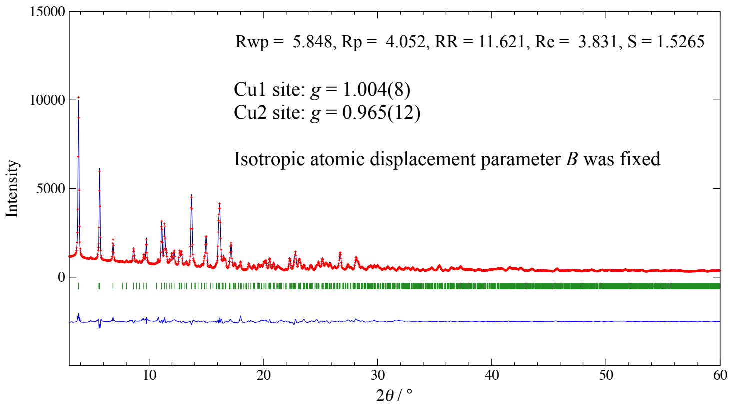

Supplementary Figure 1: Synchrotron XRD intensity pattern (red crosses) observed for $KCu_6AlBiO_4(SO_4)_5Cl$ at room temperature, the result of Rietveld refinement using the computer program RIETAN-FP (black solid line), and difference between the calculated and observed intensities (blue solid line). The green vertical bars indicate the position of Bragg reflection peaks.

Supplementary Table 1: Structure information of $KCu_6AlBiO_4(SO_4)_5Cl$

No site mixing. Isotropic atomic displacement parameter B was fixed.

Space group *P*4/*ncc* (No.130), *a* = 9.8248(9) Å and *c* = 20.5715(24) Å.

Atom	x	y	z	B	site
Cu1	0.4799(6)	0.2676(9)	0.8970(3)	0.5	16g
Cu2	0	0	0	0.5	8d
Al	0.25	0.25	0.7003(16)	1.0	4c
Bi	0.25	0.25	0.5126(2)	0.5	4c
K	0.75	0.25	0.75	1.0	4a
Cl	0.25	0.25	0.8195(12)	1.0	4c
S1	0.5690(17)	0.9838(17)	0.8505(8)	1.0	16g
S2	0.75	0.25	0	1.0	4b
O1	0.4398(47)	0.9613(34)	0.8313(20)	1.0	16g
O2	0.6921(37)	0.8618(31)	0.8051(18)	1.0	16g
O3	0.6049(43)	0.1189(44)	0.8386(22)	1.0	16g
O4	0.5820(49)	0.9579(45)	0.9165(17)	1.0	16g
O5	0.6595(33)	0.3297(38)	0.9584(19)	1.0	16g
O6	0.3431(50)	0.3796(49)	0.9561(16)	1.0	16g

Supplementary Note 2: Magnetic susceptibility and Magnetization

As shown in Supplementary Fig. 2, a zero-field-cooled/field-cooled ZFC/FC divergence is not observed. In addition, the *M-H* curves show no hysteresis at 1.8 K and 20 K. These results indicate the absence of spin glass behavior, thus suggesting that this compound with an ideal 2D J_1-J_2-J_3 SKL has no site-mixing magnetic defects.

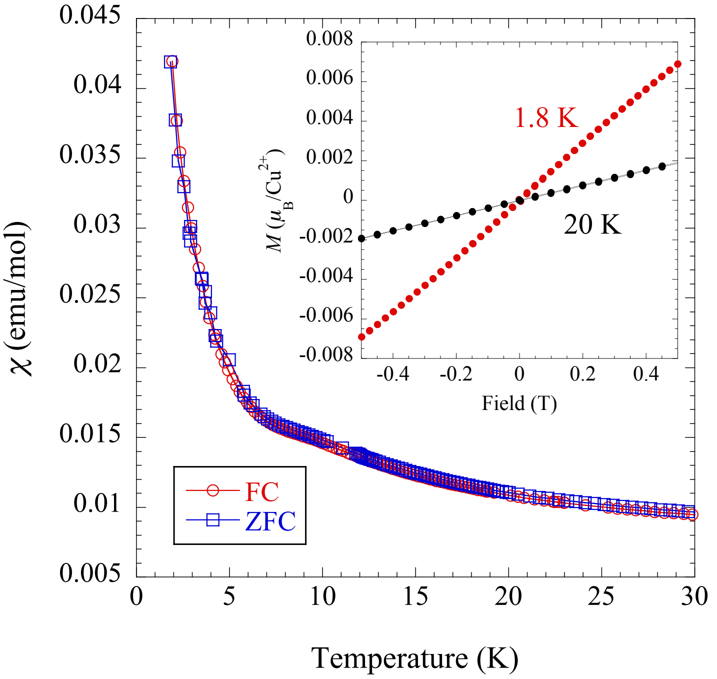

Supplementary Figure 2: Magnetic susceptibilities measured under zero-field-cooled (ZFC) and field-cooled (FC) conditions at 100 Oe. The inset plot shows magnetisation measured at 1.8 and 20 K.

Supplementary Note 3: μSR EXPERIMENT

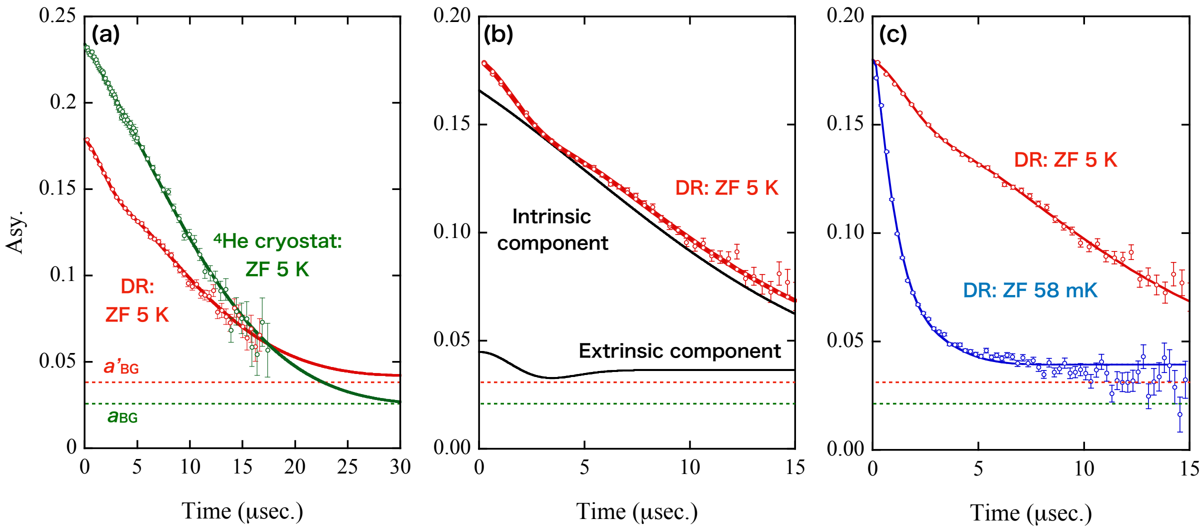

Supplementary Figure 3: (a) ZF-μSR spectra measured at 5 K using a dilution refrigerator (DR) and ^{4}He cryostat. The thick lines behind the data are fitted curves described by Supplementary Eq. 1 and 2. (b) ZF-μSR spectrum measured at 5 K using a DR. The fitted curve can be decomposed into an intrinsic and extrinsic component. (c) ZF-μSR spectra measured at 5 K and 58 mK using a DR. The 58mK data is also fitted by Supplementary Eq. 2.

The spectra were collected in the temperature range from 58 mK to 300 K using a dilution refrigerator (DR) and ^{4}He cryostat. We have carried out both μSR measurements using powder sample of same synthesis batch. The difference of the initial asymmetries (0.18 for DR and 0.23 for ^{4}He cryostat) in Supplementary Fig. 3 may be attributed to the different sample weights. The completely different environments in a dilution refrigerator and ^{4}He cryostat, the wall of the vacuum vessel, number of windows, thickness of the sample holder, etc., may be one of the causes of this difference.

Supplementary Fig. 3 shows the ZF-μSR spectra which are measured at 5 K. The ZF-spectrum measured using the ^{4}He cryostat is well fitted by

$$a(t) = a_0\, G_{\mathrm{KT}}(\Delta_{intrinsic}, t)\, \mathrm{e}^{-\lambda t} + a_{\mathrm{BG}} \quad \text{(Supplementary Eq. 1)}$$

where a_0 is an intrinsic asymmetry and a_{BG} is a constant background that represents muons that missed the sample; therefore, the initial asymmetry is the sum of a_0 and a_{BG}. a_{BG} is determined by the size, shape and density of the sample and the sample environment, and thus there is a discrepancy in the value of a_{BG} between measurements using a ^{4}He cryostat and dilution refrigerator. $G_{\mathrm{KT}}(\Delta, t)$ is the static Gaussian Kubo-Toyabe function [Supplementary Reference 3], $\Delta_{intrinsic}$ (= 0.056) is the distribution width of the internal field, and λ (= 0.049) is the muon spin relaxation rate. However, Supplementary Eq. (1) cannot be used in case of the ZF-spectra measured using the DR. These data are well-described by the following function:

$$a(t) = a_{intrinsic}\, G_{\mathrm{KT}}(\Delta_{intrinsic}, t)\, \mathrm{e}^{-\lambda t} + a_{extrinsic}\, G_{\mathrm{KT}}(\Delta_{extrinsic}, t) + a'_{\mathrm{BG}} \quad \text{(Supplementary Eq. 2)}$$

where $G_{\mathrm{KT}}(\Delta_{extrinsic}, t)$ is the relaxation function for the second component. We fitted the 5 K DR data using Supplementary Eq. (2), and obtain $a_{intrinsic}$ = 0.133, $a_{extrinsic}$ = 0.013, a'_{BG} = 0.034, $\Delta_{extrinsic}$ = 0.28, however, the intrinsic distribution width of the internal field and muon spin relaxation rate are fixed as $\Delta_{intrinsic}$ = 0.056 and λ = 0.049 (see Supplementary Fig. 3(b)). We could not trace the origin of the second component, however, we are convinced that this component is extrinsic component because the ^{4}He cryostat is simpler in structure than the DR. As shown in Supplementary Fig. 3(c), the component seems to show no temperature dependence, indicating this is static component. The local static field is estimated to be B_{loc} = 5.8 G (= $\Delta_{extrinsic}/\gamma_\mu$). Thus, the static local field can fully be decoupled even at the fields of 50 G. Therefore, the extrinsic component can be regarded as background for the weak LF (50 G) μSR spectra, and the total background a_{BG} is defined as $a_{extrinsic} + a'_{\mathrm{BG}}$ = 0.047. In fact, as shown in Fig. 3(b), the background increases at 50 G.

The 58mK data is also fitted by Eq. (S2). The relaxation rates λ at 58 mK is 0.759 μs^{-1}. If this spectrum is due to static magnetism, the internal field can be estimated as λ/γ_μ (γ_μ is the muon gyromagnetic ratio), and thus it should be less than 10 G. However, as shown in Fig. 3(c), the relaxation is clearly observed, even in the LF at 0.395 T, which is evidence for the fluctuation of Cu^{2+} electron spins without spin ordering/freezing.

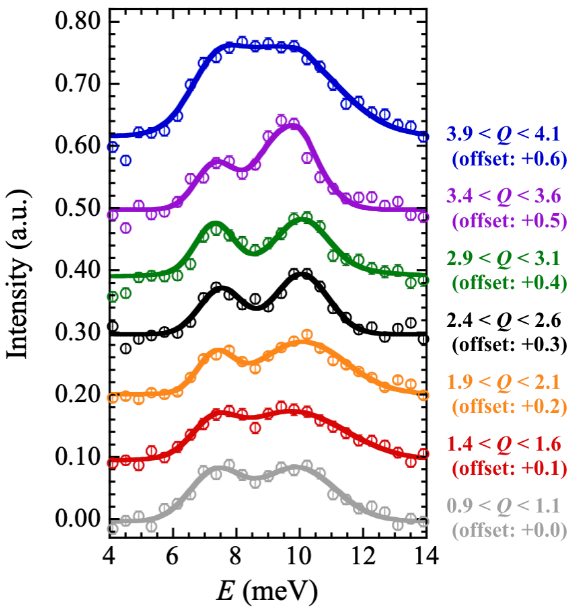

Supplementary Figure 4: Energy dependence of the scattering integrated over several ranges in Q measured at 5 K (HRC). The linear baseline is subtracted from each data.

Supplementary Note 4: INS MEASUREMENT

As shown in Supplementary Fig. 4, the E-dependence of the INS intensity observed using HRC which is subtracted linear baseline from each data can be fitted well by a multi-Gaussian fitting. Energy dependence of the scattering integrated over Q in the range 2.9 Å^{-1} < Q < 3.1 Å^{-1}, 3.4 Å^{-1} < Q < 3.6 Å^{-1}, and 3.9 Å^{-1} < Q <

4.1 Å^{-1} can be decomposed into three Gaussian components. The other cuts can be fitted two Gaussian functions.

The spectra were observed at 0.3 K and 30 K using AMATERAS. The streak-like signals are observed at least down to 0.2 meV, and the intensity of that increase with continuously without the energy gap. The signal is developed at low temperatures.

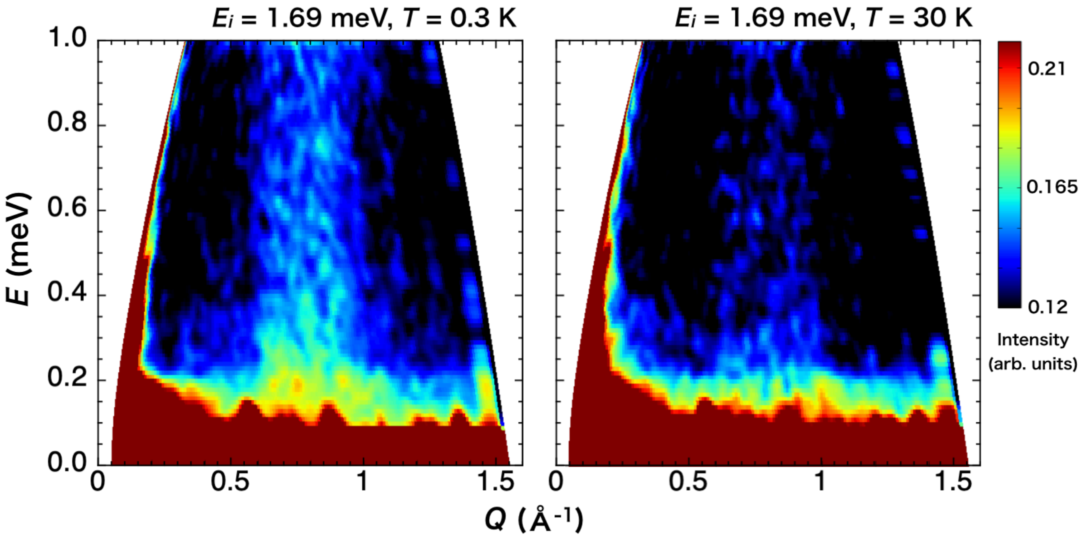

Supplementary Figure 5: INS spectra at 0.3 K (left) and 30 K (right) observed using AMATERAS with incident neutron energy of 1.69 meV.

Supplementary Note 5: Theoretical study

We verify the validity for our determined exchange interactions of $KCu_6AlBiO_4(SO_4)_5Cl$, that is, J_1 = 135 K, J_2 = 162 K and J_3 = 115 K. We calculated the magnetic susceptibility using the orthogonalized FTL method [Supplementary Reference 4] for a 24-site cluster of the J_1-J_2-J_3 SKL. Supplementary Fig. 5 shows the calculated results at five different ratios with respect to J_1, J_2 and J_3 and the experimental result, where, J_{av} defined as $(J_1 + J_2 + J_3) / 3$ is kept constant at 137 K in order to keep the Weiss temperature constant. We can see that the result at $J_1 : J_2 : J_3$ = 1 : 0.85 : 1.20 corresponding to our determined parameters is the best match for the experimental one. Moreover, we also succeeded in reproducing the magnetisation curve of $KCu_6AlBiO_4(SO_4)_5Cl$ with the J_1-J_2-J_3 SKL with these parameters (see Fig.5(b) in the main text). Therefore, we determined that the exchange interactions of $KCu_6AlBiO_4(SO_4)_5Cl$ are J_1 = 135 K, J_2 = 162 K and J_3 = 115 K.

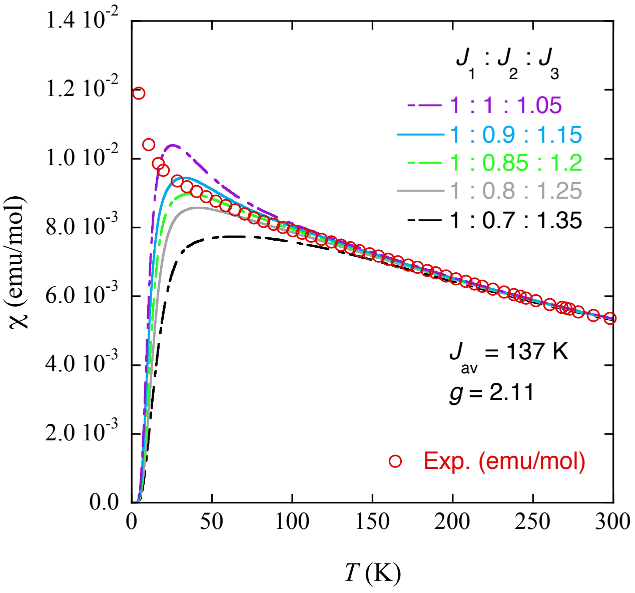

Supplementary Figure 6: Results of the magnetic susceptibility for the 24-site cluster of the J_1-J_2-J_3 SKL of using the orthogonalized FTL method and the experimental result. Here, J_{av} is defined as $(J_1 + J_2 + J_3) / 3$.

Supplementary References

1. V. I. Popova, V. A. Popov, N. S. Rudashevsky, S. F. Glavatskikh, V. O. Polyakov, A. F. Bushmakin, Zap Vses Mineral Obshch **116**, 358 (1987).

2. F. Izumi & K. Momma, Solid State Phenom **130**, 15 (2007).

3. R. S. Hayano, Y. J. Uemura, J. Imazato, N. Nishida, T. Yamazaki and R. Kubo, Physical Review B **20**, 850 (1979).

4. K. Morita & T. Tohyama, Phys. Rev. Research **2**, 013205 (2020).
